# Sepsis and antimicrobial stewardship: two sides of the same coin

**DOI:** 10.1136/bmjqs-2019-009445

**Published:** 2019-04-24

**Authors:** Fidelma Fitzpatrick, Carolyn Tarrant, Vida Hamilton, Fiona M Kiernan, David Jenkins, Eva M Krockow

**Affiliations:** 1 Clinical Microbiology, RCSI, Dublin, Ireland; 2 Clinical Microbiology, Beaumont Hospital, Dublin, Ireland; 3 Department of Health Sciences, University of Leicester, Leicester, UK; 4 Department of Anaesthesia & Intensive Care, University College Hospital, Waterford, Waterford, Ireland; 5 Department of Anaesthesia and Intensive Care Medicine, RCSI, Dublin, Ireland; 6 Department of Clinical Microbiology, University Hospitals of Leicester NHS Trust, Leicester, UK; 7 Department of Health Sciences, University of Leicester, Leicester, UK

**Keywords:** antibiotic management, clinical practice guidelines, critical care, emergency department, health policy

## Introduction

Sepsis and antimicrobial stewardship programmes coexist in tension, as they can appear to have apparently opposing messages around antimicrobial prescribing. In the era of increasing antimicrobial resistance (AMR), there is a need for greater alignment between sepsis and antimicrobial stewardship governance and management programmes. Antimicrobial therapy is an essential part of sepsis management with a focus on time-dependent recognition and resuscitation pathways.^1^ Sepsis is a clinical diagnosis, and delay to first-dose antimicrobial is associated with increasing mortality.^1^ To avoid potential unintended consequences from inappropriate antimicrobial prescribing, including increased AMR and healthcare-associated infections such as *Clostridioides difficile* infection, antimicrobial stewardship strategies including de-escalation protocols and stopping antimicrobials in non-infective cases should be a fundamental component of sepsis quality improvement initiatives.^2^ Perceived tensions remain, however, between managing sepsis and effective antimicrobial stewardship, and these perceptions are likely to be heightened by media reporting of the issues.^3^ The purpose of this viewpoint is to discuss these tensions, and suggest that a change in mindset is required with an integrated understanding of sepsis and AMR as two sides of the same coin.

## Media framing of sepsis and AMR

Media framing of sepsis and AMR has been identified as influencing public expectation of antimicrobial prescribing as well as health professionals’ perceptions of optimal prescribing strategies. Sepsis media reports tend to use well-recognised triggers that increase public interest. These include emotive personal narratives that commonly centre on young patients, and which identify immediate solutions that are within the power of individual health professionals and the public (eg, increased awareness/recognition). In contrast, AMR is framed as a vague threat affecting future patients and involving multiple actors, often under the ‘One Health’ umbrella.^4^ Individual responsibility is diffused by presenting AMR as a global responsibility, which shares conceptual features with a ‘problem of many hands’,^5 6^ and the types of solutions identified are longer-term ones requiring mobilisation of governments and policy change. Given human decision makers’ tendency to prefer short-term rewards over delayed rewards—a concept referred to as hyperbolic discounting—this aspect of media reporting does little to encourage individual responsibility or motivation to optimise antimicrobial use. Another feature of both media reporting and public health campaigns is that sepsis and AMR are rarely presented together, resulting in a lack of understanding of how the issues are interrelated. An overview of typical media frames is provided in table 1.

**Table 1:** Overview of how different problem aspects of sepsis and antimicrobial resistance (AMR) are typically framed in media reports (original table summarising findings from previous research)^3^

Problem aspect	Sepsis	AMR
Geographical scope	National/Local	Global
Problem definition	Individual patient safety	Public health issue
Immediacy of threat	Immediate threat	Future threat
Concreteness of threat	Concrete threat	Vague threat
Emotive nature of problem	Emotional	Abstract
Complexity of problem	Straightforward	Complicated
Responsibility for problem	Responsibility with individuals	Responsibility with government
Solution to problem	Behavioural solution	Biological/technical solution

High-profile stories of preventable deaths from sepsis can lead people to potentially overestimate the frequency that an infection could be sepsis.^7^ Behavioural economists attribute this effect to the so-called recognition heuristic, in which mere exposure to a particular message heightens its perceived significance.^8^ Less frequently considered is the impact of media reports on prescriber behaviour, although an increasing number of qualitative investigations into antimicrobial prescribing decisions identified the fear of missing sepsis as a major concern and significant cause of defensive prescribing practice.^9–13^ A recent literature review highlighted different contributors to prescribers’ anxieties, including their strong sense of ‘duty of care’ to current patients and an emotional investment in their recovery.^9^ Additional factors are concerns about complaints and litigation in case of bad patient outcomes, damage to the professional reputation or reprimands by superiors for missing organisational targets. Junior doctors especially may struggle with the often poorly defined antimicrobial prescribing responsibilities. These anxieties may be compounded by sepsis media reports, which could promote higher levels of risk aversion and inappropriate prescribing decisions of doctors.

## Balancing sepsis and antimicrobial stewardship goals in practice

The first step in sepsis management pathways is recognition of patients with sepsis; however, this itself is challenging. Sepsis is a subjective clinical rather than a laboratory diagnosis and recognition can be difficult, especially early in the clinical presentation when symptoms are non-specific and laboratory results are pending. This means clinicians must act before the results of investigations are available. While the majority of experienced clinicians report confidence in applying sepsis definitions, only a minority successfully identified sepsis case vignettes in an experimental test of their diagnostic accuracy.^14^ In the context of uncertainty, clinicians must act, weighing up the risks of failing to treat sepsis against overdiagnosis, overtreatment and the associated risk of increasing AMR. The visibility of the sepsis threat involving acutely unwell patients and the emphasis on time-dependent protocols bring to the forefront the immediate risks posed by sepsis and the need for urgent action by individual healthcare staff.

Current antimicrobial stewardship guidance aims to strike a balance between promptly managing suspected infection and avoiding antimicrobial overuse. Recommendations include the development of bespoke guidelines based on local AMR epidemiology to standardise antimicrobial prescribing, a blended learning approach to antimicrobial stewardship education, standardised antimicrobial consumption surveillance and close liaison with the diagnostic laboratory.^15^ While antimicrobial stewardship guidance stresses a rapid response to sepsis symptoms through administering broad-spectrum empiric antimicrobials, it equally focuses on subsequent patient review, switching to a more targeted treatment or stopping antimicrobials. There is, however, evidence of reluctance to alter antimicrobial therapy in a patient with sepsis due to a lack of conclusive research findings regarding the safety and efficacy of this approach,^16^ and the lack of perceived immediacy of the problem of AMR. This has been confirmed by findings that prescribers focus on avoiding immediate risks of mortality and morbidity by avoiding changing ‘a winning team’ (ie, a seemingly successful treatment approach).^17^

Sepsis and antimicrobial stewardship initiatives are frequently implemented by different teams and individuals within healthcare organisations, resulting in lack of alignment of goals and activities. Hospitals in the USA were mandated to report compliance with the ‘SEP-1’ sepsis bundle from late 2015.^18 19^ This bundle included administration of broad-spectrum antimicrobials within 3 hours of sepsis onset. In addition to concern regarding the sepsis definitions used,^20^ setting time-based targets for antimicrobial administration can adversely affect antimicrobial stewardship efforts. Previous time-based US targets for pneumonia, which included financial compensation for timely administration of antimicrobials, had negative consequences for antimicrobial stewardship. In a study conducted in Detroit, Michigan, the accuracy of pneumonia diagnoses decreased and overall antimicrobial use increased because of implementation of the pneumonia target.^21^ In Ireland, efforts to align sepsis and antimicrobial stewardship include recommendations that sepsis governance structures include antimicrobial stewardship representation, and presentation of sepsis process and outcome measurements alongside balancing measures which include antimicrobial consumption data and *C. difficile* infection rates.^22^ In England, the Sepsis Commissioning for Quality and Innovation (CQUIN) recently introduced national quality or CQUIN indicators. These included time-based sepsis targets (namely, timely identification and treatment of sepsis in emergency departments and acute inpatient settings) linked with antimicrobial stewardship targets (antimicrobial review; reduction in antimicrobial consumption per 1000 admissions).^23^ These targets have attached financial incentives, but it remains to be seen whether this improves sepsis management and/or antimicrobial prescribing.

## Sepsis and antimicrobial stewardship: two sides of the same coin

While both the drive to tackle sepsis and antimicrobial stewardship initiatives represent efforts to implement evidence into practice and improve the quality of healthcare, the respective healthcare messages need to be more integrated. Sepsis and antimicrobial stewardship cannot be discussed in isolation and should be portrayed as two sides of the same coin. The terminology and framing used in relation to sepsis and AMR in the media and messages promoted through public health campaigns also need to be considered. Achieving an appropriate balance requires reframing AMR as an immediate problem with consequences for individual practitioners and patients. Furthermore, inappropriate antimicrobial prescribing should be framed as a public health threat and a patient safety issue. This should include highlighting positive effects of more targeted prescribing, including the reduction of individual side effects such as hospital-acquired, multidrug-resistant infections. Following principles from behavioural economics (eg, decision-making heuristics, framing effects and hyperbolic discounting) when formulating these health messages could help to balance perceptions of sepsis and antimicrobial stewardship and could help to achieve a ‘recognition of necessity’ to change prescribing approaches. Within healthcare organisations, there is a need for local alignment of sepsis and antimicrobial stewardship programmes, with coordinated responsibilities and consistent messages. In the future, new technologies enabling real-time, near-patient diagnosis of sepsis and identification of antimicrobial susceptibilities of the associated infection will be crucial to reducing uncertainty and the need for empirical prescribing decisions prone to bias. It will therefore require combined efforts from clinicians including sepsis and antimicrobial stewardship experts and social science researchers to influence policy makers, journalists and the public.
